# Validated screening tools to identify common mental disorders in perinatal and postpartum women in India: a systematic review and meta-analysis

**DOI:** 10.1186/s12888-021-03190-6

**Published:** 2021-04-20

**Authors:** Gracia Fellmeth, Siân Harrison, Charles Opondo, Manisha Nair, Jennifer J. Kurinczuk, Fiona Alderdice

**Affiliations:** 1 National Perinatal Epidemiology Unit, Nuffield Department of Population Health, University of Oxford, Old Road Campus, Oxford, OX3 7LF UK; 2 School of Nursing and Midwifery, Queen’s University Belfast, Belfast, BT9 7BL Northern Ireland

**Keywords:** Common mental disorder, Screening, Low- and middle-income country (LMIC), Validation, Systematic review, India, Perinatal

## Abstract

**Background:**

Perinatal common mental disorders are associated with significant adverse outcomes for women and their families, particularly in low- and middle-income settings. Early detection through screening with locally-validated tools can improve outcomes.

**Methods:**

We searched MEDLINE, Embase, PsycINFO, Global Health, Cochrane Library, Web of Science and Google Scholar for articles on the validation of screening tools for common mental disorders in perinatal women in India, with no language or date restrictions. Quality was assessed using the QUADAS-2 tool. We used bivariate and hierarchical summary receiver operating characteristic models to calculate pooled summary estimates of sensitivity and specificity. Heterogeneity was assessed by visualising the distance of individual studies from the summary curve.

**Results:**

Seven studies involving 1003 women were analysed. All studies assessed the validity of the Edinburgh Postnatal Depression Scale (EPDS) in identifying perinatal depression. No validation studies of any other screening tools were identified. Using a common threshold of ≥13 the EPDS had a pooled sensitivity and specificity of 88·9% (95%CI 77·4–94·9) and 93·4 (95%CI 81·5–97·8), respectively. Using optimal thresholds (range ≥ 9 to ≥13) the EPDS had a pooled sensitivity and specificity of 94·4% (95%CI 81·7–98·4) and 90·8 (95%CI 83·7–95·0), respectively.

**Conclusion:**

The EPDS is psychometrically valid in diverse Indian settings and its use in routine maternity care could improve detection of perinatal depression. Further research is required to validate screening tools for other perinatal common mental disorders in India.

**Supplementary Information:**

The online version contains supplementary material available at 10.1186/s12888-021-03190-6.

## Background

Globally, mental health disorders are among the greatest contributors to maternal morbidity and mortality, with low- and middle-income countries (LMIC) carrying the greatest burden [1]. Common mental disorders (CMD) refer to depressive and anxiety disorders, which represent the most prevalent categories of mental health disorders [2]. CMD experienced in the perinatal period (during pregnancy or the first twelve months post-partum) are associated with significant and potentially long-term consequences for women and their families [3]. In LMIC in particular, infants of mothers with mental disorders have a higher risk of adverse physical, cognitive and behavioural outcomes including low birthweight, stunting, infectious illnesses and delayed development [3, 4]. Meta-analyses from LMIC have reported pooled prevalence estimates of 19% for perinatal depression [5], and 34 and 26% for antenatal and postnatal anxiety [6], respectively, although estimates from individual settings vary according to population characteristics, socio-cultural factors and methodological variations.

Early detection and treatment of perinatal CMD is essential to minimise adverse effects and improve outcomes for women and their families [7, 8]. However, stigma around mental health disorders, competing clinical priorities, a lack of expertise, no systematic means of detection and poor mental health service infrastructure mean that in many LMICs women with mental disorders remain unidentified, unsupported and untreated. Screening for CMD symptoms offers a systematic and efficient means of identifying women likely to have mental health disorders [7, 8]. Screening tools must be validated locally against a ‘gold standard’ diagnostic interview prior to use to assess their validity, establish the appropriate diagnostic thresholds and ensure their cultural appropriateness and acceptability [9]. Without formal validation, the reliability of screening tools in a setting or population different from that in which the tool was developed is not guaranteed. Screening with an inappropriately high threshold risks under-detection of women with CMD, while a threshold that is too low risks creating high levels of demand, placing strain on already over-burdened health systems [10]. Furthermore, the lack of validation brings into question the reliability of prevalence estimates from a variety of settings, as most prevalence studies rely on the use of screening tools to determine caseness. A previous review of screening tools for CMD highlighted a shortage of validation studies from LMIC, with particular gaps in screening tools for anxiety disorders [11].

In India, an estimated 18 and 22% of women experience antenatal [12] and postnatal [13] depression, respectively, while perinatal anxiety has not been systematically studied. Stigma towards mental disorders remains widespread nationally, and women living in socio-economic deprivation, those experiencing intimate partner violence and those with low societal status are at particularly high risk of perinatal mental disorders [14, 15]. Although there have been initiatives to improve maternal health, there has been less focus on maternal mental health and services in this area are lacking [14]. There is no screening initiative recommended for routine use in maternity services [13]. Without a systematic synthesis of Indian validation studies there remains uncertainty around which screening tools might be suitable for use in perinatal populations in India. Improving the detection of perinatal mental disorders is key to better CMD management and to achieving many of the United Nation’s Sustainable Development Goals [16]. The current review fills an important literature gap by synthesising the current evidence about validated screening tools for perinatal CMD in India, thereby providing evidence-based data for screening initiatives that can improve women’s wellbeing across India.

## Methods

### Search strategy and selection criteria

References for this systematic review and meta-analysis were identified through searches of MEDLINE, Embase, PsycINFO, Global Health, the Cochrane library and Web of Science using a combination of MeSH and free-text terms pertaining to perinatal status, common mental disorders, validation and India. The full search strategy is detailed in the Additional file 1. In order to maximise inclusivity, no date or language restrictions were used. Non-indexed journal articles and grey literature were identified through searches of the National Database of Indian Medical Journals (MedIND), the World Health Organisation (WHO), the United Nations Children’s Fund, the Government of India Ministry of Health and Family Welfare and *Google Scholar*. Reference lists of relevant studies were manually searched to identify further eligible studies. Studies of perinatal women of any age, in any trimester of pregnancy and up to twelve months post-partum and living in India were included. Studies of Indian nationals living abroad were excluded. Studies of any design were included if they assessed the performance of a CMD screening tool against a diagnostic interview. Studies of screening tools assessing quality of life or maternal-infant bonding were excluded. Studies which compared one screening tool to another (rather than to a diagnostic interview) were included but this limitation was reflected in the study’s quality assessment rating. No studies were excluded on the basis of quality. The primary outcome was measures of psychometric validity including sensitivity, specificity, area under the receiver operating characteristic (ROC) curve (AUC) and optimal cut-off thresholds of the screening tool. Secondary outcomes were the acceptability and ease of administration of the tool, and CMD prevalence as determined by diagnostic interviews. The final search was conducted on 16 April 2020. This study was registered with PROSPERO (CRD42019153711)

### Screening, data extraction and risk of bias assessment

This review was carried out in accordance with the Preferred Reporting Items for Systematic Review and Meta-Analysis (PRISMA) guidelines [17]. Two reviewers (GF, SH) independently screened titles and abstracts using *Covidence* software to identify potentially eligible studies [18]. Full-texts of these studies were retrieved and screened independently by the same reviewers. Disagreements were resolved by discussion with a third reviewer (FA). For included studies, two reviewers (GF, SH) independently extracted data about study design and setting, participant characteristics, screening tool, diagnostic interview and results using a standardised, pre-piloted form (Additional file 2). Discrepancies were resolved through discussion with a third reviewer (FA). Study authors were contacted by email to retrieve missing information; up to two reminder emails were sent if no response was received to the first. Risk of bias was assessed using the Quality of Diagnostic Accuracy Studies-2 (QUADAS-2) tool which assesses patient selection (recruitment and inclusion criteria), index test and reference standard (how tests were conducted and interpreted), and the flow and timing (flow of participants through the study and time interval between tests) [19]. We added questions to assess for: (i) use of a clear and concise perinatal time period; (ii) adequate sample size; (iii) adherence to the WHO protocol for translation and adaptation of instruments into local languages [20]; and (iv) qualifications of diagnostic interviewers (Additional file 3). A minimum of 150 participants was considered a good sample size [21–23]. Two reviewers (GF, SH) assessed risk of bias for each included study and discussed discrepancies with a third reviewer (FA).

### Data analysis

A narrative synthesis of results was conducted to provide an overview of included studies. Meta-analysis was conducted for studies which validated the same screening tool and reported sufficient data [24]. Data on true positives (TP), true negatives (TN), false positives (FP) and false negatives (FN) was used to plot the sensitivity and specificity of each study in a scatterplot, illustrating the location of data points and any relationship between sensitivity and specificity seen across studies. A pooled summary estimate of sensitivity and specificity was calculated by fitting two hierarchical models that account for the threshold effect of diagnostic studies: the bivariate model and the hierarchical summary receiver operating characteristic (HSROC) model [25]. For each study included in meta-analysis, the TP, TN, FP and FN data were extracted for (i) the optimal cut-off and (ii) a common (standard) threshold. The optimal cut-off was the threshold which was identified by original studies as maximising sensitivity and specificity in that particular setting. This optimal threshold varied across studies. In addition, data was extracted on a common threshold: we used the standard cut-off of ≥13 and applied the sensitivity and specificity at this threshold from original studies. The reason for including both optimal and common thresholds was that the former assesses the performance of the EPDS using locally-determined, ideal cut-offs, while the latter provides a comparison of the EPDS performance across different settings using a standardised cut-off. HSROC plots were drawn to illustrate the pooled estimate of sensitivity and specificity (summary point) using optimal and common fixed thresholds along with 95% confidence regions [25]. Subgroup analyses were planned a-priori according to perinatal status, geographic region, language, and study quality. Heterogeneity in test accuracy was assessed by visualising the distance of individual studies from the summary ROC curve, and publication bias was explored using a scatterplot of the inverse square effective sample size (ESS) versus the diagnostic odds ratio (DOR) [24]. All analyses were conducted using STATA version 14.2 [26].

## Results

**Fig. 1:**
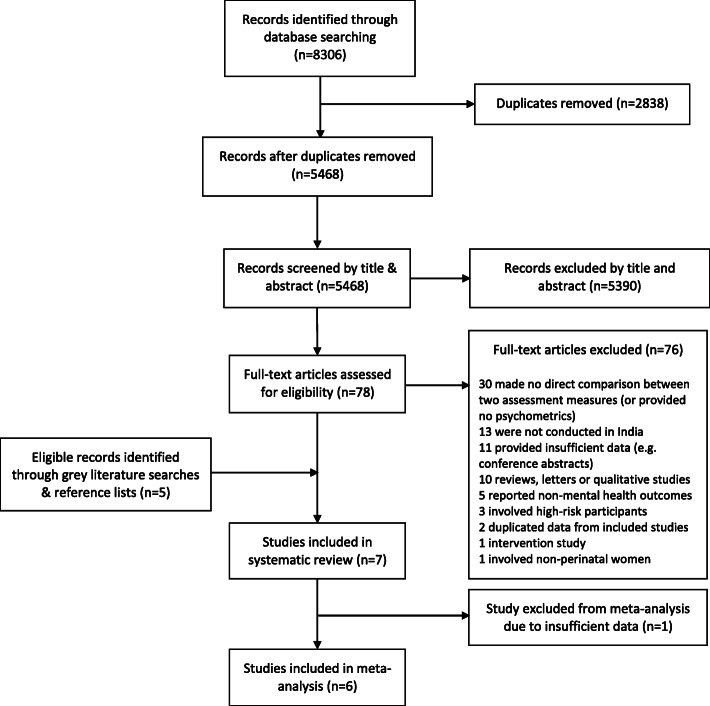
Study selection

We identified 8306 publications through electronic database searches, of which 2838 were duplicates (Fig. 1). Of the 5468 records screened by title and abstract, 78 were included in full-text screening. Of these full-texts, 76 were excluded for reasons listed in Fig. 1. Five additional journal articles were identified through grey literature searches. Seven studies [27–33] met the criteria for inclusion in the review and six [27–32] were included in meta-analysis; it was not possible to include the seventh study [33] because this study did not report participant numbers. Characteristics of included studies are shown in Table 1. Studies were all cross-sectional and included data for over 1003 rural and urban perinatal women in total (one study did not report participant numbers). Settings were geographically diverse, including Assam in the Northeast [31]; Gujarat [28], Maharasthra [32] and Goa [33] in the West; Madhya Pradesh in central India [30]; and Karnataka [29] and Tamil Nadu [27] in the South. Screening tool languages included Hindi [30], Assamese [31], Gujarati [28], Kannada [29], Konkani [33], Marathi [32], and Tamil [27]. Four studies included post-partum women [27, 28, 31, 32], two included pregnant women [29, 30], and one study did not report the timing of assessment [33].

**Table 1:** Characteristics of studies included in the systematic review

Study	Setting; rural/urban	Study design	Sample	Condition	Screening tool	Diagnostic interview	Language	Optimal cut-off	Sens	Spec	Prevalence^1^	Other
Benjamin (2005)	Tamil Nadu (South); rural	CS	129 post-partum women	Depression	EPDS	CIS-R	Tamil	≥9	94.1%	90.2%	13.2%	AUC 0.921 At cut-off ≥13: sens 64.7%; spec 96.4%
Desai (2011)	Gujarat (West); mixed	CS	200 post-partum women (0–12 months)	Depression	EPDS	Psychiatrist interview	Gujarati	≥11	100%	98%	12.5%	AUC 0.999; PPV 89.3; NPV 100 At cut-off ≥13: sens 84%; spec 100%
Fernandes (2011)	Karnataka (Southwest); rural	CS	194 pregnant women (3rd trimester)	Depression	EPDS K10	MINI-Plus	Kannada	EPDS: ≥13 K10: ≥6	EPDS: 100% K10: 100%	EPDS: 84.9% K10: 81.3%	14.3%	EPDS: AUC 0.95 (95% CI 0.92–0.97); PPV 52.0; NPV 99.0 K10: AUC 0.95 (95% CI 0.92–0.98); PPV 48.0; NPV 99.0
Joshi (2020)	Madhya Pradesh (central); mixed	CS	100 pregnant women (12–36 weeks)	Depression	EPDS	PHQ-9	Hindi	≥10	65.4%	79.7%	–	AUC 0.7346 (95% CI 0.562–0.796) At cut-off ≥13: sens 78.6%; spec 75.6%
Kalita (2008)	Assam (Northeast); mixed	CS	100 post-partum women (6 weeks)	Depression	EPDS	ICD-10 criteria	Assamese	≥13	88.9%	85.4%	18%	AUC NR; PPV 57.14; NPV 97.22
Khapre (2017)	Maharashtra (West); rural	CS	280 post-partum women (6 weeks to 6 months)	Depression	EPDS	Semi-structured interview	Marathi	≥13	93.8%	94.9%	–	AUC 0.968 (95% CI 0.947–0.989) At cut-off ≥13: sens 93.8%; spec 94.9%
Patel (2002)	Goa (West); NR	CS	NR	Depression	EPDS	CIS-R	Konkani	11 or 12	92%	85%	–	AUC NR

*Abbreviations*: *AUC* area under the receiver operating curve, *CI* confidence interval, *CIS-R* Clinical Interview Schedule (Revised), *CS* cross sectional, *EPDS* Edinburgh Postnatal Depression Scale, *ICD-10* International Classification of Diseases (10th revision), *K10* Kessler-10, *MINI* Mini-International Neuropsychiatric Interview, *NPV* negative predictive value, *NR* not reported, *PHQ-9* Patient Health Questionnaire-9, *PPV* positive predictive value, *Sens* sensitivity, *Spec* specificity. ^1^Prevalence determined using diagnostic interview

All of the included studies validated the Edinburgh Postnatal Depression Scale (EPDS) as a screening tool for depression. No studies were identified which validated any other CMD screening tool. Six studies [27–29, 31–33] validated the EPDS against a diagnostic interview and one [30] compared the EPDS to another screening tool (Patient Health Questionnaire-9; PHQ-9). Fernandes et al. (2011) simultaneously validated the EPDS and the Kessler-10 (K10) scale against a diagnostic interview [29]. All screening tools were administered verbally by healthcare workers or researchers. Diagnostic interviews used as the comparators included the Clinical Interview Schedule (Revised) (CIS-R), Mini-International Neuropsychiatric Interview (MINI-Plus) and semi-structured psychiatric interviews based on ICD-10 criteria. Optimal EPDS cut-offs identified in individual studies ranged between ≥9^27^ and ≥13 [29, 31, 32]. Sensitivity and specificity at these optimal cut-offs ranged between 65·4–100% [28, 29] and 79·7–98% [28, 30], respectively. The AUC at these optimal thresholds ranged between 0·7346–0·999 [28, 30] across five studies which reported this metric. When a common threshold of ≥13 was applied across all studies, including the three studies for which this was not the optimal threshold, sensitivity and specificity of the EPDS ranged between 64·7–100% [27, 29] and 84·8–100% [28, 29], respectively.

Three studies explored the acceptability of screening tools to participating women and healthcare staff. Benjamin et al. (2005) and Desai et al. (2011) reported ease of administration of the EPDS due to its brevity and avoidance of technical terms [27, 28]. Desai et al. (2011) found that the EPDS took on average five minutes to complete, and that the tool itself, as well as the wider concept of routine screening for post-partum depression, were feasible and acceptable to participants [28]. Fernandes et al. (2011) found that in comparison to the K10, the EPDS was more difficult to administer due to its changing response options which were time-consuming to explain [29]. However, the EPDS was better than the K10 at excluding somatic symptoms of pregnancy [29]. Khapre (2017) did not directly assess acceptability to participants but reported that no linguistic ambiguity was found in the translated EPDS and that the screen took 7–10 min on average to complete [32]. The prevalence of depression as measured using diagnostic interviews was reported by four studies and ranged between 12.5% [28] and 18% [31].

**Fig. 2:**
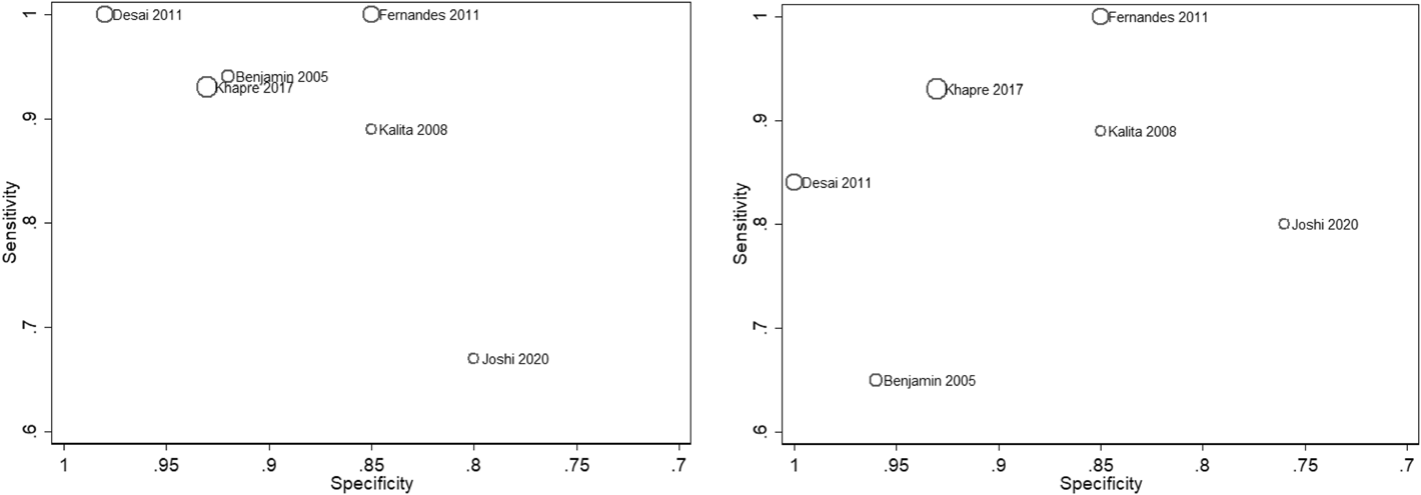
Scatterplot of sensitivity vs. specificity of the EPDS for detecting depression in each study using optimal thresholds (left panel) and common threshold of ≥13 (right panel)

**Fig. 3:**
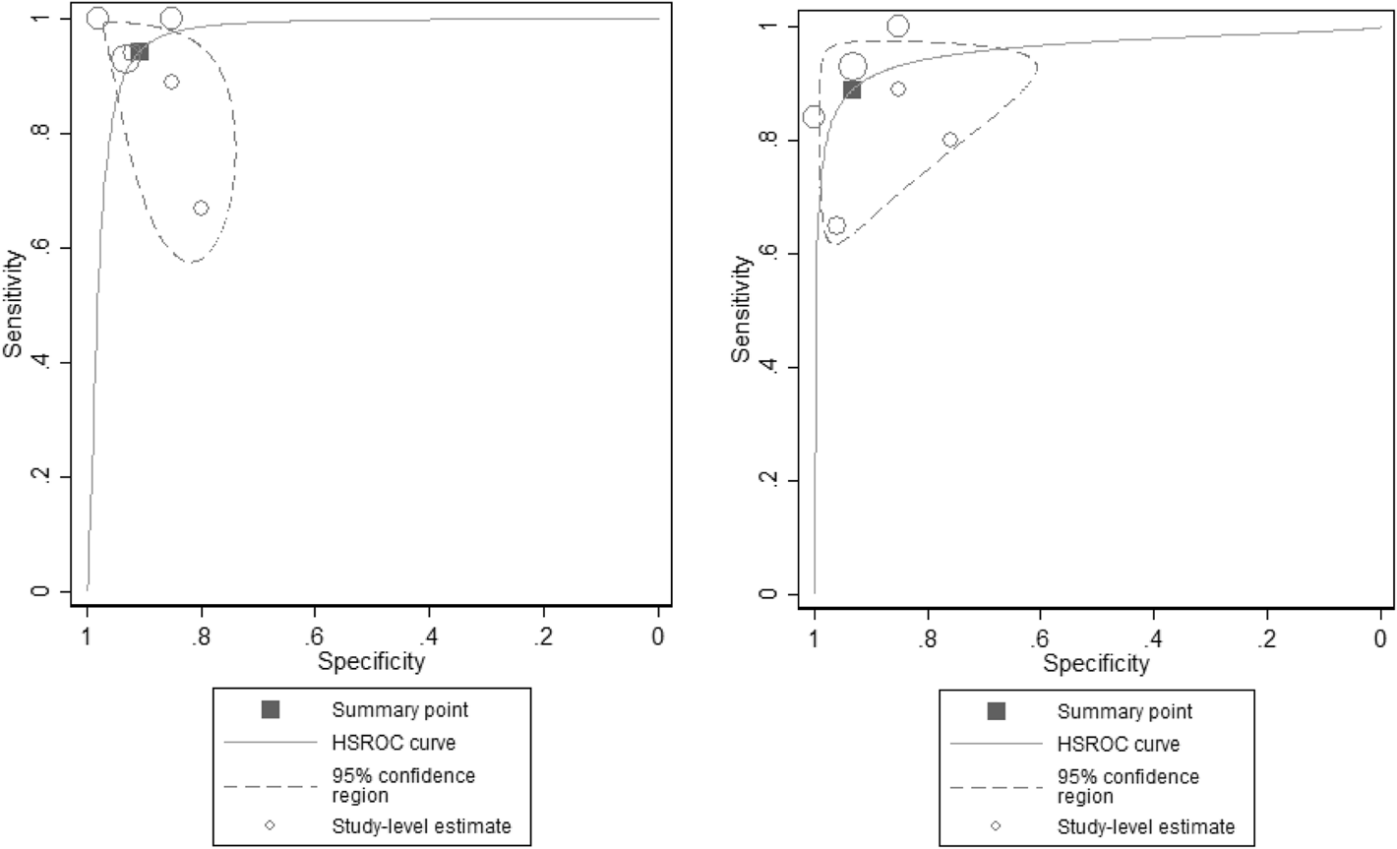
Hierarchical summary receiver operating characteristic (HSROC) curve using optimal threshold (left panel) and common threshold of ≥13 (right panel)

Six studies (1003 participants) were included in meta-analysis [27–32]. Fig. 2 shows scatterplots of the sensitivity and specificity of each study using optimal thresholds and a common threshold of ≥13. Optimal thresholds ranged from ≥9 to ≥13. Using these optimal thresholds, Desai et al. (2011) had the highest sensitivity and specificity, Joshi et al. (2020) had the lowest and the remaining studies were located in between. At a threshold of ≥13, Desai et al. (2011), Khapre et al. (2017) and Fernandes et al. (2011) had the highest sensitivities and specificities, followed by Kalita et al. (2008), Benjamin et al. (2005) and Joshi et al. (2020). Figure 3 shows the HSROC curves along with summary points of pooled sensitivity and specificity and 95% confidence regions using optimal and common thresholds. Using optimal thresholds, the pooled sensitivity estimate was 94·4% (95% CI 81·7–98·4) and the pooled specificity estimate was 90·8 (95% CI 83·7–95·0). Using a common threshold of ≥13, the pooled sensitivity estimate was 88·9% (95% CI 77·4–94·9) and the pooled specificity estimate was 93·4 (95% CI 81·5–97·8). The small number of studies available for meta-analysis precluded subgroup analyses. There was no evidence of publication bias, as shown by the lack of association between the inverse square roots of the effective sample sizes of the included studies and their estimated diagnostic odds ratios (Additional file 4). Study quality varied by domains assessed (Table 2). Two studies were considered to be at low risk of bias across all domains [28, 29], and two studies were considered to be at high risk of bias across one or more domains [30, 31].

**Table 2:**
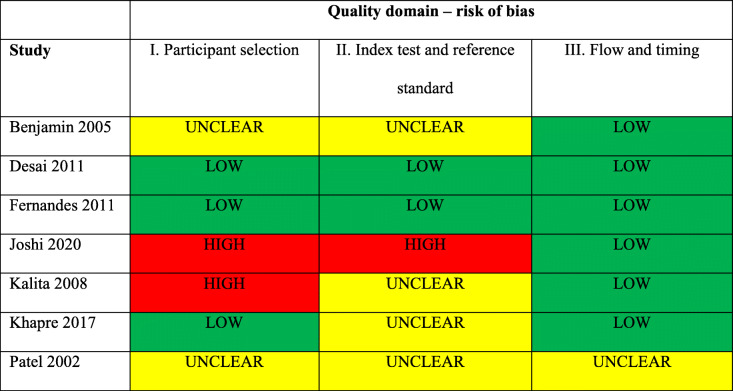
Risk of bias (quality) of studies included in review as assessed using QADAS-2

low risk of bias;  unclear risk of bias;  high risk of bias

## Discussion

This systematic review and meta-analysis synthesises data from validation studies of screening tools for perinatal CMDs in India. Seven studies were identified, all of which assessed the validity of the EPDS in identifying perinatal depression. Although the review aimed to assess validation studies of *any* CMD screening tools, no validation studies of any mental disorders other than depression, nor of any screening tools other than the EPDS, were identified. Studies included in the review reported good psychometric performance of the EPDS with optimal thresholds ranging from ≥9 to ≥13. Using optimal thresholds, the pooled sensitivity and specificity were 94·4% and 90·8%, respectively. When a common EPDS threshold of ≥13 was applied across all studies, the pooled sensitivity and specificity were not substantially different at 88·9% and 93·4%, respectively. Overall, these findings suggest that the EPDS is a psychometrically valid tool that can reliably detect women with perinatal depression across diverse settings and languages in India.

Our pooled estimates suggest that EPDS sensitivity is maximised when an optimal threshold, identified through the formal validation of the tool in the local context and population, is applied. Validity in one setting or population does not imply validity elsewhere, and especially in a country as culturally, linguistically and socially diverse as the Indian subcontinent, local validation to suit local needs is the ideal practice [11, 34–36]. Previous studies have highlighted differences in optimal thresholds across locations. In a review of EPDS validity across LMIC settings, optimal thresholds ranged from 4 to 12, with sensitivity and specificity ranging from 69·7–100% and 36·1–97%, respectively [10]. These differences are likely the combined results of several factors including cultural differences in manifestations of depression, population characteristics and methodological differences such as mode of assessment [34, 37].

To assess the EPDS’ performance at a fixed threshold, we selected a cut-off of ≥13 which is one of the most commonly-used thresholds globally [37]. This cut-off was also recommended by the original EPDS authors as being indicative of ‘probable depression’ [38]. At a common threshold of ≥13 the pooled sensitivity of the EPDS decreased, but only marginally, from 94·4% to 88·9%, while the pooled specificity increased marginally from 90·8% to 93·4%. Among individual studies, there was variability in EPDS performance at this cut-off: Fernandes et al. (2011) reported 100% sensitivity among pregnant women in rural Karnataka while Benjamin et al. (2005) reported a sensitivity of 64·7% among postpartum women in rural Tamil Nadu [27, 29]. Language and cultural factors may have played a role in these differences, along with the different perinatal stage (pregnant vs. post-partum) of participants. These differences highlight the importance of local validation and adaptation of the threshold whenever possible. However, we acknowledge that resources and capacity to conduct local validation studies using robust methodology may not always be available in LMIC settings, where existing services are already stretched. When local validation is not feasible, our results suggest that a standardised threshold, the validity of which has been demonstrated in a similar population and setting, may provide a next-best alternative with only a marginal decrease in test performance.

The acceptability of an instrument to respondents and healthcare workers is a crucial aspect of screening yet often remains overlooked. A screening tool that is difficult or time-consuming will have limited utility and is unlikely to be sustainable in routine care. The three studies in our review which explored the acceptability of the EPDS all reported ease-of-use and good acceptability [27–29]. However, the only study to compare the EPDS to another screening tool found that the alternative, the K10, was quicker and more straightforward to administer [29]. The EPDS is generally considered to have good acceptability across diverse settings [34, 37, 39, 40]. However, challenges have been reported in some settings. In a study of migrant and refugee women in Thailand, participants and staff found the EPDS difficult to use and time-consuming, rendering it unsuitable for the local context [41]. In the UK, a review of the acceptability of the EPDS found that although the tool was generally well-accepted postnatally, women found some EPDS items difficult and questioned the cultural specificity of the tool [42]. Our review found that the prevalence of perinatal depression in various states in India using diagnostic interviews ranged from 12·5 to 18%. This range is lower than previous estimates derived from screening tools, which tend to over-estimate prevalence [12, 13]. Our prevalence range may provide a more accurate estimate of the true burden of perinatal depression in India.

The quality of included studies varied. While several studies scored well across all domains, some weak areas were seen across studies. Few studies explicitly reported following the standardised WHO protocol for the translation and adaptation of tools [20]. Exclusion criteria were another weakness. Joshi et al. (2020) excluded women with a previous history of severe mental disorder, suicidal ideation or anti-depressant medication, and Kalita et al. (2008) excluded women with past substance misuse or on long-term medication [30, 31]. These exclusions are problematic given the correlation between these factors and perinatal depression, and may have limited the generalisability of findings to the wider community.

The general paucity of evidence around validation of screening tools for perinatal CMD in India is of concern. The existing evidence is narrow in scope, focusing entirely on perinatal depression and almost exclusively on the EPDS. Although the EPDS appears to be a psychometrically sound and generally well-accepted tool, the lack of research on alternative depression screening tools means we cannot make overall recommendations for the EPDS over other tools. Other CMDs and also common and often co-morbid with perinatal depression [6]. Studies to assess the validity of screening tools for these conditions are an urgent research priority. Five of the seven studies in this review were identified through grey literature searches. Although these were journal publications, they appeared in non-indexed journals and were therefore not retrieved by our medical database searches. The absence of these studies from the most commonly used databases for reviewing the medical literature highlights the importance of looking beyond indexed journals in order to avoid missing important and relevant studies.

India has seen a steady decline in maternal mortality in recent years and this presents an opportunity to shift focus to addressing maternal morbidity [13]. Given the significant associations between perinatal CMD and the well-being of women, their children, families and wider society, the identification and appropriate management of perinatal CMDs must not be sidelined. The perinatal period is a time of increased contact between women and healthcare services, providing an ideal opportunity for screening and intervention [36]. In practice, however, this opportunity is all too often missed and perinatal CMD too often remains undetected and untreated. If implemented with sensitivity, screening for perinatal CMD can help to identify women in need of support and treatment [42, 43]. Current gaps in the evidence, specifically around the validity of screening tools for anxiety disorders, hinder the practice of screening perinatal women. More research in this area, including testing the acceptability of instruments to the local population, is essential to guide practice and inform policy-makers on evidence-based means of identifying women with CMD at an early stage.

To our knowledge, this is the first review of validation studies for perinatal CMD in India. Strengths include broad search terms, a comprehensive database search without language or date restrictions and a thorough grey literature search. Throughout the title and abstract screening stages, we applied an inclusive approach: any study referring to more than one assessment tool for CMD in the title or abstract was included in full-text screening even when it was not labelled as a validation study in order to check for any relevant data. Additional data from authors enabled us to calculate sensitivity and specificity for all studies at a common threshold of ≥13. A limitation of the review is its restriction to CMD, so that other mental health disorders such as psychotic disorders were excluded.

## Conclusion

CMDs experienced during pregnancy and the postpartum period all too often remain undetected and untreated, causing suffering, distress and disability for affected women and their families. Screening tools which have been validated and are appropriate to the local setting and population are important to improve the identification of women with perinatal depression and anxiety disorders. Evidence about the validity of screening tools for perinatal CMD in India is limited: existing studies focus exclusively on perinatal depression and exclusively on the EPDS. The EPDS performed well as a screening tool for perinatal depression and in the absence of local validation to establish an optimal threshold, a standardised cut-off from similar settings could be considered as a next best alternative. Comparisons of the EPDS with other depression screening tools are needed to establish which tool is most appropriate in Indian settings and guide practitioners and policy-makers. The lack of research on perinatal anxiety screening tools represents a significant evidence gap which must be addressed. Increasing the detection of perinatal mental disorders is key to better supporting women’s mental wellbeing and, ultimately, improving maternal morbidity in India.

## Supplementary Information

**Additional file 1:.** Search strategies.

**Additional file 2:.** Data extraction form.

**Additional file 3:.** Quality assessment (QADAS-2) tool.

**Additional file 4:.** Deeks funnel plot asymmetry test for publication bias.

## Data Availability

The dataset supporting the conclusions of this article is included within the article and its additional files.

## Abbreviations

AUC: Area under the receiver operating characteristic curve
CI: Confidence intervals
CIS-R: Clinical Interview Schedule, Revised
CMD: Common mental disorder
DOR: Diagnostic odds ratio
EPDS: Edinburgh Postnatal Depression Scale
ESS: Effective sample size
FN: False negative
FP: False positive
HSROC: Hierarchical summary receiver operating characteristic
ICD-10: International Classification of Diseases, 10th revision
K-10: Kessler-10
LMIC: Low- and middle-income country
MINI: Mini-International Neuropsychiatric Interview
NPV: Negative predictive value
PHQ-9: Patient Health Questionnaire-9
PPV: Positive predictive value
PRISMA: Preferred Reporting Item for Systematic Reviews and Meta-Analyses
QADAS-2: Quality of Diagnostic Accuracy Studies-2
ROC: Receiver operating characteristic
TN: True negative
TP: True positive
WHO: World Health Organization
