# Supplementary material for: Validated screening tools to identify common mental disorders in perinatal and postpartum women in India: a systematic review and meta-analysis
Source: BMC Psychiatry. 2021 Apr 20;21:200. doi: 10.1186/s12888-021-03190-6 (PMC8056564; doi:10.1186/s12888-021-03190-6)
Supplement: Supplementary file 1 — Additional file 1:. Search strategies. [file 12888_2021_3190_MOESM1_ESM.docx]

**Additional file 1. Search strategies**

Medline (Ovid)

1 Prenatal Care/

2 Pregnancy/

3 Maternal Health/

4 (antenatal or ante-natal or antepartum or ante-partum or birth or childbirth or maternal or mother or natal or perinatal or peri-natal or peripartum or peri-partum or postnatal or post-natal or postpartum or post-partum or pregnant or pregnancy or prenatal or pre-natal or puerperal or trimester or women).ti

5 1 or 2 or 3 or 4

6 mental disorders/ or exp anxiety disorders/ or exp "bipolar and related disorders"/ or exp mood disorders/ or exp "trauma and stressor related disorders"/

7 Depression/

8 Anxiety/

9 (adjustment or affective or anxiety or anxious or bipolar or common mental disorder or CMD or compulsive or depression or depressed or depressive or distress* or GAD or mood or obsessive or OCD or panic or phobia or posttrauma or posttraumatic or post-trauma or post-traumatic or PTSD or psychiatric or psychological or psychosis or stress* or trauma).ti.

10 6 or 7 or 8 or 9

11 5 and 10

12 Depression, Postpartum/

13 (antenatal or ante-natal or antepartum or ante-partum or birth or childbirth or maternal or mother or natal or perinatal or peri-natal or peripartum or peri-partum or postnatal or post-natal or postpartum or post-partum or pregnant or pregnancy or prenatal or pre-natal or puerperal or trimester or women) adj5 (adjustment or affective or anxiety or anxious or bipolar or common mental disorder or CMD or compulsive or depression or depressed or depressive or distress* or GAD or mood or obsessive or OCD or panic or phobia or posttrauma or posttraumatic or post-trauma or post-traumatic or PTSD or psychiatric or psychological or psychosis or stress* or trauma).ti,ab.

14 11 or 12 or 13

15 (assessment or checklist or diagnosis or diagnostic or identification or identify or index or instrument or interview or inventory or questionnaire or reliable or reliability or scale or score or scoring or screen or screening or sensitive or sensitivity or specific or specificity or standard or standardised or standardized or survey or test or tool or validation or validity).ti,ab.

16 (India or Indian or Andhra or Arunachal or Assam* or Bengal* or Bhalodi or Bhilai or Bihar or Bodo or Chhattisgarh or Dogru or Garo or Goa or Gondii or Gujarat* or Haryana or Himachal or Hind* or Jammu or Jharkhand or Kannada or Karnataka or Kashmir* or Kerala or Khandesh or Khasi or Kontani or Kuraku or Madhya or Maharashtra or Maithili or Malayalam or Manipur* or Marathi or Meitei or Meghalaya or Mizoram or Munderi or Nadu or Nagaland or Nepali or Odia or Odisha or Pradesh or Punjab* or Rajasthan or Sanskrit or Santoli or Sindhi or Sikkim or Tamil or Telangana or Telugu or Tripura or Tulu or Urdu or Uttar).af.

17 14 and 15 and 16

Embase (Ovid)

1 Prenatal Care/

2 Pregnancy/

3 Maternal Health/

4 (antenatal or ante-natal or antepartum or ante-partum or birth or childbirth or maternal or mother or natal or perinatal or peri-natal or peripartum or peri-partum or postnatal or post-natal or postpartum or post-partum or pregnant or pregnancy or prenatal or pre-natal or puerperal or trimester or women).ti.

5 1 or 2 or 3 or 4

6 mental disorders/ or exp anxiety disorders/ or exp "bipolar and related disorders"/ or exp mood disorders/ or exp "trauma and stressor related disorders"/

7 Depression/

8 Anxiety/

9 (adjustment or affective or anxiety or anxious or bipolar or common mental disorder or CMD or compulsive or depression or depressed or depressive or distress* or GAD or mood or obsessive or OCD or panic or phobia or posttrauma or posttraumatic or post-trauma or post-traumatic or PTSD or psychiatric or psychological or psychosis or stress* or trauma).ti.

10 6 or 7 or 8 or 9

11 5 and 10

12 Depression, Postpartum/

13 (antenatal or ante-natal or antepartum or ante-partum or birth or childbirth or maternal or mother or natal or perinatal or peri-natal or peripartum or peri-partum or postnatal or post-natal or postpartum or post-partum or pregnant or pregnancy or prenatal or pre-natal or puerperal or trimester or women) adj5 (adjustment or affective or anxiety or anxious or bipolar or common mental disorder or CMD or compulsive or depression or depressed or depressive or distress* or GAD or mood or obsessive or OCD or panic or phobia or posttrauma or posttraumatic or post-trauma or post-traumatic or PTSD or psychiatric or psychological or psychosis or stress* or trauma).ti,ab.

14 11 or 12 or 13

15 (assessment or checklist or diagnosis or diagnostic or identification or identify or index or instrument or interview or inventory or questionnaire or reliable or reliability or scale or score or scoring or screen or screening or sensitive or sensitivity or specific or specificity or standard or standardised or standardized or survey or test or tool or validation or validity).ti,ab.

16 (India or Indian or Andhra or Arunachal or Assam* or Bengal* or Bhalodi or Bhilai or Bihar or Bodo or Chhattisgarh or Dogru or Garo or Goa or Gondii or Gujarat* or Haryana or Himachal or Hind* or Jammu or Jharkhand or Kannada or Karnataka or Kashmir* or Kerala or Khandesh or Khasi or Kontani or Kuraku or Madhya or Maharashtra or Maithili or Malayalam or Manipur* or Marathi or Meitei or Meghalaya or Mizoram or Munderi or Nadu or Nagaland or Nepali or Odia or Odisha or Pradesh or Punjab* or Rajasthan or Sanskrit or Santoli or Sindhi or Sikkim or Tamil or Telangana or Telugu or Tripura or Tulu or Urdu or Uttar).af.

17 14 and 15 and 16

PsycINFO (Ovid)

1 Prenatal Care/

2 Pregnancy/

3 Mothers/

4 (antenatal or ante-natal or antepartum or ante-partum or birth or childbirth or maternal or mother or natal or perinatal or peri-natal or peripartum or peri-partum or postnatal or post-natal or postpartum or post-partum or pregnant or pregnancy or prenatal or pre-natal or puerperal or trimester or women).ti.

5 1 or 2 or 3 or 4

6 mental disorders/ or exp anxiety disorders/ or exp bipolar disorder/ or exp mood disorders/ or exp "stress and trauma related disorders"/

7 Depression/

8 Anxiety/

9 (adjustment or affective or anxiety or anxious or bipolar or common mental disorder or CMD or compulsive or depression or depressed or depressive or distress* or GAD or mood or obsessive or OCD or panic or phobia or posttrauma or posttraumatic or post-trauma or post-traumatic or PTSD or psychiatric or psychological or psychosis or stress* or trauma).ti.

10 6 or 7 or 8 or 9

11 5 and 10

12 Postpartum Depression/

13 (antenatal or ante-natal or antepartum or ante-partum or birth or childbirth or maternal or mother or natal or perinatal or peri-natal or peripartum or peri-partum or postnatal or post-natal or postpartum or post-partum or pregnant or pregnancy or prenatal or pre-natal or puerperal or trimester or women) adj5 (adjustment or affective or anxiety or anxious or bipolar or common mental disorder or CMD or compulsive or depression or depressed or depressive or distress* or GAD or mood or obsessive or OCD or panic or phobia or posttrauma or posttraumatic or post-trauma or post-traumatic or PTSD or psychiatric or psychological or psychosis or stress* or trauma).ti,ab.

14 11 or 12 or 13

15 (assessment or checklist or diagnosis or diagnostic or identification or identify or index or instrument or interview or inventory or questionnaire or reliable or reliability or scale or score or scoring or screen or screening or sensitive or sensitivity or specific or specificity or standard or standardised or standardized or survey or test or tool or validation or validity).ti,ab.

16 (India or Indian or Andhra or Arunachal or Assam* or Bengal* or Bhalodi or Bhilai or Bihar or Bodo or Chhattisgarh or Dogru or Garo or Goa or Gondii or Gujarat* or Haryana or Himachal or Hind* or Jammu or Jharkhand or Kannada or Karnataka or Kashmir* or Kerala or Khandesh or Khasi or Kontani or Kuraku or Madhya or Maharashtra or Maithili or Malayalam or Manipur* or Marathi or Meitei or Meghalaya or Mizoram or Munderi or Nadu or Nagaland or Nepali or Odia or Odisha or Pradesh or Punjab* or Rajasthan or Sanskrit or Santoli or Sindhi or Sikkim or Tamil or Telangana or Telugu or Tripura or Tulu or Urdu or Uttar).af.

17 14 and 15 and 16

Global Health (Ovid)

1 Prenatal Care/

2 Pregnancy/

3 Mothers/

4 Postnatal period/

5 Postpartum period/

6 (antenatal or ante-natal or antepartum or ante-partum or birth or childbirth or maternal or mother or natal or perinatal or peri-natal or peripartum or peri-partum or postnatal or post-natal or postpartum or post-partum or pregnant or pregnancy or prenatal or pre-natal or puerperal or trimester or women).ti.

7 1 or 2 or 3 or 4 or 5 or 6

8 mental disorders/ or exp bipolar disorder/ or exp post-traumatic stress disorder/ or exp depression/ or exp emotional disturbances/ or exp neuroses/ or exp obsessive-compulsive disorder/ or exp phobias/

9 Depression/

10 Anxiety/

11 (adjustment or affective or anxiety or anxious or bipolar or common mental disorder or CMD or compulsive or depression or depressed or depressive or distress* or GAD or mood or obsessive or OCD or panic or phobia or posttrauma or posttraumatic or post-trauma or post-traumatic or PTSD or psychiatric or psychological or psychosis or stress* or trauma).ti.

12 8 or 9 or 10 or 11

13 7 and 12

14 (antenatal or ante-natal or antepartum or ante-partum or birth or childbirth or maternal or mother or natal or perinatal or peri-natal or peripartum or peri-partum or postnatal or post-natal or postpartum or post-partum or pregnant or pregnancy or prenatal or pre-natal or puerperal or trimester or women) adj5 (adjustment or affective or anxiety or anxious or bipolar or common mental disorder or CMD or compulsive or depression or depressed or depressive or distress* or GAD or mood or obsessive or OCD or panic or phobia or posttrauma or posttraumatic or post-trauma or post-traumatic or PTSD or psychiatric or psychological or psychosis or stress* or trauma).ti,ab.

15 13 or 14

16 (assessment or checklist or diagnosis or diagnostic or identification or identify or index or instrument or interview or inventory or questionnaire or reliable or reliability or scale or score or scoring or screen or screening or sensitive or sensitivity or specific or specificity or standard or standardised or standardized or survey or test or tool or validation or validity).ti,ab.

17 (India or Indian or Andhra or Arunachal or Assam* or Bengal* or Bhalodi or Bhilai or Bihar or Bodo or Chhattisgarh or Dogru or Garo or Goa or Gondii or Gujarat* or Haryana or Himachal or Hind* or Jammu or Jharkhand or Kannada or Karnataka or Kashmir* or Kerala or Khandesh or Khasi or Kontani or Kuraku or Madhya or Maharashtra or Maithili or Malayalam or Manipur* or Marathi or Meitei or Meghalaya or Mizoram or Munderi or Nadu or Nagaland or Nepali or Odia or Odisha or Pradesh or Punjab* or Rajasthan or Sanskrit or Santoli or Sindhi or Sikkim or Tamil or Telangana or Telugu or Tripura or Tulu or Urdu or Uttar).af.

18 15 and 16 and 17

Web of Science

1 TI=(antenatal OR ante-natal OR antepartum OR ante-partum OR birth OR childbirth OR maternal OR mother OR natal OR perinatal OR peri-natal OR peripartum OR peri-partum OR postnatal OR post-natal OR postpartum OR post-partum OR pregnant OR pregnancy OR prenatal OR pre-natal OR puerperal OR trimester OR women)

2 TI=(adjustment OR affective OR anxiety OR anxious OR bipolar OR common mental disorder OR CMD OR compulsive OR depression OR depressed OR depressive OR distress* OR GAD OR mood OR obsessive OR OCD OR panic OR phobia OR posttrauma OR posttraumatic OR post-trauma OR post-traumatic OR PTSD OR psychiatric OR psychological OR psychosis OR stress* OR trauma)

3 #2 AND #1

4 TS=((antenatal OR ante-natal OR antepartum OR ante-partum OR birth OR childbirth OR maternal OR mother OR natal OR perinatal OR peri-natal OR peripartum OR peri-partum OR postnatal OR post-natal OR postpartum OR post-partum OR pregnant OR pregnancy OR prenatal OR pre-natal OR puerperal OR trimester OR women) NEAR/5 (adjustment OR affective OR anxiety OR anxious OR bipolar OR “common mental disorder” OR CMD OR compulsive OR depression OR depressed OR depressive OR distress* OR GAD OR mood OR obsessive OR OCD OR panic OR phobia OR posttrauma OR posttraumatic OR post-trauma OR post-traumatic OR PTSD OR psychiatric OR psychological OR psychosis OR stress* OR trauma))

5 #4 OR #3

6 TS=(assessment OR checklist OR diagnosis OR diagnostic OR identification OR identify OR index OR instrument OR interview OR inventory OR questionnaire OR reliable OR reliability OR scale OR score OR scoring OR screen OR screening OR sensitive OR sensitivity OR specific OR specificity OR standard OR standardised OR standardized OR survey OR test OR tool OR validation OR validity)

7 ALL=(India OR Indian OR Andhra OR Arunachal OR Assam* OR Bengal* OR Bhalodi OR Bhilai

OR Bihar OR Bodo OR Chhattisgarh OR Dogru OR Garo OR Goa OR Gondii OR Gujarat* OR Haryana OR Himachal OR Hind* OR Jammu OR Jharkhand OR Kannada OR Karnataka OR Kashmir* OR Kerala OR Khandesh OR Khasi OR Kontani OR Kuraku OR Madhya OR Maharashtra OR Maithili OR Malayalam OR Manipur* OR Marathi OR Meitei OR Meghalaya OR Mizoram OR Munderi OR Nadu OR Nagaland OR Nepali OR Odia OR Odisha OR Pradesh OR Punjab* OR Rajasthan OR Sanskrit OR Santoli OR Sindhi)

8 ALL=(Sikkim OR Tamil OR Telangana OR Telugu OR Tripura OR Tulu OR Urdu OR Uttar)

9 #8 OR #7

10 #9 AND #6 AND #5

Cochrane

1 MeSH descriptor: [Prenatal Care] this term only

2 MeSH descriptor: [Pregnancy] this term only

3 MeSH descriptor: [Maternal Health] this term only

4 (antenatal OR ante-natal OR antepartum OR ante-partum OR birth OR childbirth OR maternal OR mother OR natal OR perinatal OR peri-natal OR peripartum OR peri-partum OR postnatal OR post-natal OR postpartum OR post-partum OR pregnant OR pregnancy OR prenatal OR pre-natal OR puerperal OR trimester OR women):ti

5 #1 or #2 or #3 or #4

6 MeSH descriptor: [Mental Disorders] this term only

7 MeSH descriptor: [Anxiety Disorders] explode all trees

8 MeSH descriptor: [Bipolar and Related Disorders] explode all trees Top of Form

Bottom of Form

9 MeSH descriptor: [Mood Disorders] explode all trees

10 MeSH descriptor: [Trauma and Stressor Related Disorders] explode all trees

11 #6 or #7 or #8 or #9 or #10

Bottom of Form

12 MeSH descriptor: [Depression] this term only Bottom of Form

13 MeSH descriptor: [Anxiety] this term only Top of Form

Bottom of Form

14 (adjustment OR affective OR anxiety OR anxious OR bipolar OR common mental disorder OR CMD OR compulsive OR depression OR depressed OR depressive OR distress* OR GAD OR mood OR obsessive OR OCD OR panic OR phobia OR posttrauma OR posttraumatic OR post-trauma OR post-traumatic OR PTSD OR psychiatric OR psychological OR psychosis OR stress* OR trauma):ti

15 Bottom of Form

1. 1#11 or #12 or #13 or #14Top of Form

16 16 #5 and #15

17 MeSH descriptor: [Depression, Postpartum] this term only

18 ((antenatal OR ante-natal OR antepartum OR ante-partum OR birth OR childbirth OR maternal OR mother OR natal OR perinatal OR peri-natal OR peripartum OR peri-partum OR postnatal OR post-natal OR postpartum OR post-partum OR pregnant OR pregnancy OR prenatal OR pre-natal OR puerperal OR trimester OR women) adj5 (adjustment OR affective OR anxiety OR anxious OR bipolar OR common mental disorder OR CMD OR compulsive OR depression OR depressed OR depressive OR distress* OR GAD OR mood OR obsessive OR OCD OR panic OR phobia OR posttrauma OR posttraumatic OR post-trauma OR post-traumatic OR PTSD OR psychiatric OR psychological OR psychosis OR stress* OR trauma)):ti,ab,kw

19 #16 or #17 or #18

20 (assessment OR checklist OR diagnosis OR diagnostic OR identification OR identify OR index OR instrument OR interview OR inventory OR questionnaire OR reliable OR reliability OR scale OR score OR scoring OR screen OR screening OR sensitive OR sensitivity OR specific OR specificity OR standard OR standardised OR standardized OR survey OR test OR tool OR validation OR validity):ti,ab,kw

Bottom of Form

21 (India OR Indian OR Andhra OR Arunachal OR Assam* OR Bengal* OR Bhalodi OR Bhilai OR Bihar OR Bodo OR Chhattisgarh OR Dogru OR Garo OR Goa OR Gondii OR Gujarat* OR Haryana OR Himachal OR Hind* OR Jammu OR Jharkhand OR Kannada OR Karnataka OR Kashmir* OR Kerala OR Khandesh OR Khasi OR Kontani OR Kuraku OR Madhya OR Maharashtra OR Maithili OR Malayalam OR Manipur* OR Marathi OR Meitei OR Meghalaya OR Mizoram OR Munderi OR Nadu OR Nagaland OR Nepali OR Odia OR Odisha OR Pradesh OR Punjab* OR Rajasthan OR Sanskrit OR Santoli OR Sindhi OR Sikkim OR Tamil OR Telangana OR Telugu OR Tripura OR Tulu OR Urdu OR Uttar)

22 #19 and #20 and #21
