# Supplementary material for: Validated screening tools to identify common mental disorders in perinatal and postpartum women in India: a systematic review and meta-analysis
Source: BMC Psychiatry. 2021 Apr 20;21:200. doi: 10.1186/s12888-021-03190-6 (PMC8056564; doi:10.1186/s12888-021-03190-6)
Supplement: Supplementary file 2 — Additional file 2:. Data extraction form. [file 12888_2021_3190_MOESM2_ESM.docx]

**Additional file 2. Data extraction form**

| First author & publication year |  |
| --- | --- |
| Citation |  |
| **Setting** |  |
| Study location (e.g. town, province) |  |
| Rural or urban |  |
| Type of setting (e.g. hospital, primary care, community) |  |
| Study design (e.g. cohort, cross-sectional) |  |
| **Participants** |  |
| Pregnant or post-partum |  |
| Timing: trimester of pregnancy/wks post-partum |  |
| Recruitment method |  |
| Any inclusion/exclusion criteria |  |
| **Screening tool** |  |
| Condition |  |
| Screening tool(s) used |  |
| Mode of administration (e.g. verbal, self-completed) |  |
| Language |  |
| **Diagnostic assessment** |  |
| Interview schedule used |  |
| Diagnosis (e.g. Major Depressive Episode) |  |
| Interviewer qualification |  |
| Timing of assessment relative to screen |  |
| **Results** |  |
| No. of participants |  |
| Age of participants (e.g. mean, sd, range) |  |
| Area under the ROC curve (AUC) |  |
| Optimal cut-off for screening tool |  |
| Overall accuracy of screening tool |  |
| Sensitivity (using optimal cut-off) |  |
| Specificity (using optimal cut-off) |  |
| PPV (using optimal cut-off) |  |
| NPV (using optimal cut-off) |  |
| Any other psychometrics (e.g. Cronbach’s alpha; LR+, LR-) |  |
| Prevalence of condition using diagnostic test |  |
| Acceptability to participants |  |
| **Any other outcomes / comments**  (e.g. interviewers’ experiences; cultural considerations) |  |
