# Supplementary material for: Validated screening tools to identify common mental disorders in perinatal and postpartum women in India: a systematic review and meta-analysis
Source: BMC Psychiatry. 2021 Apr 20;21:200. doi: 10.1186/s12888-021-03190-6 (PMC8056564; doi:10.1186/s12888-021-03190-6)
Supplement: Supplementary file 3 — Additional file 3:. Quality assessment (QADAS-2) tool. [file 12888_2021_3190_MOESM3_ESM.docx]

**Additional file 3. Quality assessment (QADAS-2) tool**

| **Domain** | **Signalling questions**  (to be answered: yes/no/unclear) | **Risk of bias**  (to be answered: high/low/unclear) |
| --- | --- | --- |
| Patient selection | Was a clear definition of perinatal status provided?* | **Could the selection of patients have introduced bias?**  If all signalling questions answered yes: LOW  If any signalling question answered no: HIGH  If insufficient data to permit judgment: UNCLEAR |
|  | Was a consecutive or random sample of patients enrolled? |  |
|  | Was a case-control design avoided? |  |
|  | Did the study avoid inappropriate exclusions? |  |
|  | Was the sample size sufficient (minimum 175 participants)?* |  |
| Index test | Was the standardised WHO protocol followed (this should include forward translation; blinded back-translation; resolving of differences by committee; and pre-testing)?* | **Could the conduct of interpretation of the index test have introduced bias?**  If all signalling questions answered yes: LOW  If any signalling question answered no: HIGH  If insufficient data to permit judgment: UNCLEAR |
|  | Were the index test results interpreted without knowledge of the results of the reference standard? |  |
|  | If a threshold was used, was it pre-specified and appropriate? |  |
| Reference standard | Was the reference standard a diagnostic measure (rather than another self-reported measure/screening tool)? | **Could the reference standard, its conduct, or its interpretation have introduced bias?**  If all signalling questions answered yes: LOW  If any signalling question answered no: HIGH  If insufficient data to permit judgment: UNCLEAR |
|  | Was the reference standard administered by a suitably qualified person?* |  |
|  | Were the reference standard results interpreted without knowledge of the results of the index test? |  |
| Flow and timing | Was there an appropriate interval between index test(s) and the reference standard? | **Could the patient flow have introduced bias?**  If all signalling questions answered yes: LOW  If any signalling question answered no: HIGH  If insufficient data to permit judgment: UNCLEAR |
|  | Did all patients receive a reference standard? |  |
|  | Did all patients receive the same reference standard? |  |
|  | Were all patients included in the analysis? |  |

* Additional questions
