# Supplementary material for: Validated screening tools to identify common mental disorders in perinatal and postpartum women in India: a systematic review and meta-analysis
Source: BMC Psychiatry. 2021 Apr 20;21:200. doi: 10.1186/s12888-021-03190-6 (PMC8056564; doi:10.1186/s12888-021-03190-6)

**Additional file 4. Deeks funnel plot asymmetry test for publication bias**

Deeks’ funnel plot asymmetry test for publication bias using (a) optimal threshold (left) and (b) common threshold (right)


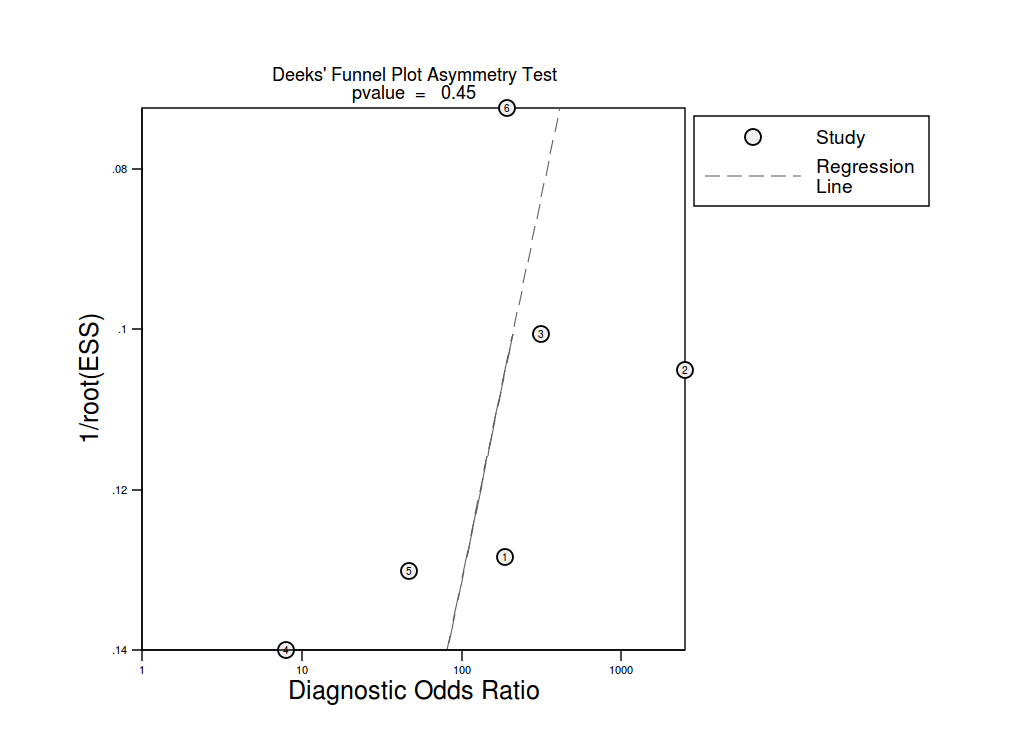

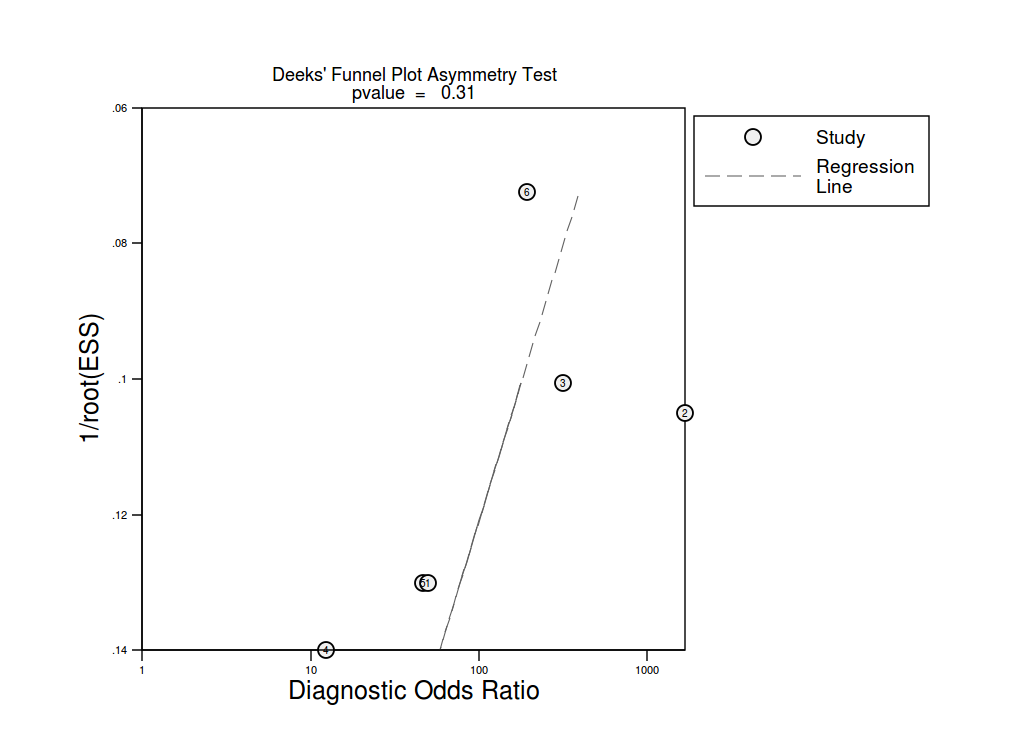

Supplement: Supplementary file 4 — Additional file 4:. Deeks funnel plot asymmetry test for publication bias. [file 12888_2021_3190_MOESM4_ESM.docx]
